# The global, regional, and national disease burden of breast cancer attributable to low physical activity from 1990 to 2019: an analysis of the Global Burden of Disease Study 2019

**DOI:** 10.1186/s12966-022-01283-3

**Published:** 2022-04-02

**Authors:** Xiaolin Yin, Tongchao Zhang, Yuan Zhang, Jinyu Man, Xiaorong Yang, Ming Lu

**Affiliations:** 1 Department of Epidemiology and Biostatistics, School of Public Health, Cheeloo College of Medicine, Shandong University, Jinan, 250012 China; 2 Clinical Epidemiology Unit, Qilu Hospital of Shandong University, 107 Wenhuaxi Road, Jinan, 250012 Shandong China; 3 Clinical Research Center of Shandong University, Jinan, 250012 China

**Keywords:** Physical activity, Breast cancer, Mortality, Disability-adjusted life years, Global Burden of Disease

## Abstract

**Background:**

To assess the spatiotemporal variation in female breast cancer attributable to low physical activity (LPA) at a global scale from 1990 to 2019, which is essential to promote physical activity, as well as prevent and control breast cancer.

**Methods:**

The number of deaths and disability-adjusted life years (DALYs), and the corresponding age-standardized rates (ASMR and ASDR) of LPA-related breast cancer in 204 countries and territories from 1990 to 2019 were retrieved from the Global Burden of Disease Study 2019 to measure the related breast cancer burden by age and region. The estimated annual percentage change (EAPC) was calculated to quantify the secular trend in breast cancer burden rates.

**Results:**

From 1990 to 2019, globally, both breast cancer deaths and DALYs attributable to LPA nearly doubled, although the corresponding ASMR and ASDR decreased slightly, with EAPC of -0.46 (95% confidence interval: -0.52, -0.40) and -0.44 (95% confidence interval: -0.49, -0.39), respectively. The LPA-related breast cancer burden varied considerably across the world, with the highest-burden rates in Oceania, Tropical Latin America and Caribbean, and the fastest growth in North Africa and Middle East. The ASMR and ASDR showed a logarithmic association with the Socio-demographic Index, and a temporally upward trend in most of 204 countries regardless of the Socio-demographic Index or the ASMR in 1990.

**Conclusions:**

Despite a decline in LPA-related breast cancer burden achieved in many countries during the last 3 decades like Bermuda, Myanmar, USA and China, an increase still occurred in most of 204 countries and territories, such as Solomon Islands, Equatorial Guinea, Japan and India. The findings can bring greater awareness to the importance of promoting physical activity for the local government to control the attributable breast cancer burden.

**Supplementary Information:**

The online version contains supplementary material available at 10.1186/s12966-022-01283-3.

## Background

Breast cancer mortality rates have been declining globally in the last three decades [1], principally owing to improvements in treatment and the early detection by mammography screening [2, 3], while the number of breast cancer deaths and disability-adjusted life years (DALYs) have continued to increase [4], which perhaps can be attributed to the population growth and inadequacy of prevention strategies [2]. Keeping physically active is one of the most important recommendations presented by the World Cancer Research Fund, which suggests staying at least moderately physically active and limiting sedentary habits [5]. However, owing to the rising trends in insufficient physical activity worldwide [6], the improvement of physical activity in healthy policies needs to be strengthened, which will not only benefit breast cancer control but also reduce the risk of other causes of health burden [7].

As new evidence of the association between physical activity and risk of breast cancer becomes available and high-quality, it is important to monitor and estimate the disease burden of breast cancer attributable to low physical activity (LPA). The Global Burden of Diseases Study (GBD) 2019 has not only focused on the health burden of 369 diseases and injuries but also incorporated their associated risk factors and the relative harm they cause [8]. All the GBD data can be publicly available online, which allow decision-makers or researchers conveniently to compare the changes in effects and risk factors of different diseases. In this study, we aimed to estimate the spatiotemporal variation in breast cancer burden attributable to LPA at the global, regional and national level from 1990 to 2019, which would guide sports resource allocation and strategies developing to promote physical activity, as well as to prevent and control the breast cancer burden.

## Methods

### Study data

Annual data of LPA-related breast cancer deaths, the age-standardized mortality rate (ASMR), DALYs and the age-standardized DALY rate (ASDR) in 204 countries and territories from 1990 to 2019 were retrieved by age (5-year age groups from 15 to 94 years, and 95 + years) and region from the online Global Health Data Exchange website (http://ghdx.healthdata.org/gbd-results-tool) [9]. They were reported in the estimates with their 95% uncertainty intervals (*UI*s), which were determined using the 2.5th and 97.5th centiles of the ordered 1000 draws. All countries and territories were classified into the 5 regions (low, low-middle, middle, high-middle and high) according to the quintiles of the Socio-demographic Index (SDI), which was the geometric mean of total fertility rate under the age of 25 years, average years of education, and lag-distributed income per capita [10]. In addition, the 204 countries and territories were also divided into 21 GBD regions in terms of geography [10]. Sex was not included as a covariate in this study as GBD 2019 focused on breast cancer burden attributable to LPA only among females. This GBD-based study complied with the Guidelines for Accurate and Transparent Health Estimates Reporting recommendations (Additional File 1) and was exempted by the Ethical Board Review of Qilu Hospital, Shandong University because the GBD data were de-identified and aggregated.

### Estimation of breast cancer burden

In the GBD study, breast cancer was defined as the ICD-10 code C50-C50.9, D05-D05.9, D24-D24.9, D48.6 and D49.3, as well as the ICD-9 code 174–175.9, 217–217.8, 233.0, 238.3, 239.3, and 610–610.9. First, the mortality-to-incidence ratios were estimated using matched incidence and mortality data from cancer registries, which were used to convert incidence data of cancer registries into inputs of mortality estimation, to maximize data availability [9]. Then, the mortality data were estimated from a Cause of Death Ensemble model approach that combined multiple data from vital registration systems, cancer registries, and verbal autopsy reports. For each cancer, the sex-specific Cause of Death Ensemble models generated mortality estimates by age group, geography, and year. Finally, the mortality estimates of each cancer were scaled to keep consistent with the total mortality for all causes of death.

Breast cancer DALYs were calculated as the sum of years of life lost and years lived with disability [9]. The former was calculated by multiplying the number of estimated breast cancer deaths by the age-specific GBD life expectancy by age group. The latter was calculated by multiplying the prevalence of each sequela and the disability weights for the health state associated with this sequela, and by adding the complications caused by breast cancer treatment. The detailed input sources and relevant metadata were available via online data source tools (http://ghdx.healthdata.org/gbd-2019/data-input-sources).

### Estimation of attributable burden

Data on levels of physical activity were mainly retrieved from two standardized questionnaires, the Global Physical Activity Questionnaire and the International Physical Activity Questionnaire, as well as other questionnaires with intensity, duration and frequency of activities, among surveys of the general adult population that collected self-reported physical activity where random sampling was used [11]. The GBD study measured physical activity in the frequency, duration and intensity of activities lasting at least ten minutes at a time for all domains of life (leisure/recreation, work/household and transport) for adults older than 25. Metabolic Equivalent (MET) was introduced as the ratio of the working metabolic rate to the resting metabolic rate, to quantify the level of physical activity. Physical activity levels were divided into four categories based on the quartiles of the global total MET-minutes per week, including inactive level (< 600 MET-minutes per week), low-active level (600–3999 MET-minutes per week), moderately active level (4000–7999 MET-minutes per week) and highly active level (≥ 8000 MET-minutes per week). The proportion of each level for 21 GBD regions over time was retrieved from the Epi Visualization tool (https://vizhub.healthdata.org/epi/). The theoretical minimum risk exposure level for physical inactivity was defined as 3000–4500 MET-minutes per week based on a previous dose–response meta-analysis [12], and thus LPA was defined as < 3000 MET-minutes per week. The detailed input data were also available via online data source tools, and the part of input data not available in tools could be made available upon request.

The attributable burden was estimated using the Comparative Risk Assessment framework established previously [11], which included the following steps: including risk-outcome pairs with convincing or probable evidence; summarizing the relative risks of potential exposure based on the systematic reviews and meta-regression; estimating the exposure levels and distributions; defining the theoretical minimum risk exposure level as the exposure level associated with minimum risk determined from published trials and cohort studies; calculating the population attributable fractions (PAFs); estimating the PAFs and attributable burden for combined risk factors by considering the mediating effects. In our analysis, PAFs represented the proportion of breast cancer burden that would be reduced among a given population and in a given year if the level of physical activity in the past were at least 3000 MET-minutes per week [11]. PAF was calculated as the following model:

$${PAF}_{asgt}=\frac{{\sum }_{x=1}^{u}{RR}_{ast}\left(x\right){P}_{asgt}\left(x\right)-1}{{\sum }_{x=1}^{u}{RR}_{as}\left(x\right){P}_{asgt}\left(x\right)}$$

where ${PAF}_{asgt}$ was the PAF for breast cancer burden due to LPA for age group $a$, sex $s$, location $g$, and year $t$; ${RR}_{ast}$ was the relative risks between exposure level $x$ (from 1 to $u$) of LPA and breast cancer for age group $a$, sex $s$, and year $t$; and ${P}_{asgt}$ was the proportion of the population exposed to LPA at the level $x$, for age group $a$, sex $s$, location $g$, and year $t$. The relative risks were derived from published and unpublished primary studies or secondary meta-analysis, and had been adjusted for potential confounders by the original researchers. Finally, the breast cancer burden specifically attributable to LPA was calculated by multiplying the total breast cancer burden by PAF after considering the mediating effects, for each age group, sex, geography, and year [11].

### Statistical analysis

The deaths, ASMR, DALYs and ASDR were used to quantify the breast cancer burden attributable to LPA at global, regional, and national levels, and the estimated annual percentage change (EAPC) was introduced to measure the temporal trends of age-standardized rates (ASRs) from 1990 to 2019. A regression model, $\mathrm{ln}(ASR)=\alpha +\beta x+\varepsilon$, was fitted to the natural logarithm of ASR, where $x$ referred to the calendar year. EAPC was calculated as 100 × (exp(*β*) − 1), and its 95% confidence intervals (*CI*s) could also be calculated from the model [13, 14]. The ASR would be upward if the EAPC and its 95% *CI* were > 0, downward if they were < 0, and stable if the 95% *CI* included 0. Finally, Spearman’s correlation was used to test the association between ASR, SDI values and the EAPC estimates. All statistical analyses were conducted via software R (version 4.0.3, R core team), and data were visualized with ggplot2 and RcolorBrewer packages.

## Results

### Breast cancer deaths and ASMR attributable to LPA

**Fig. 1:**
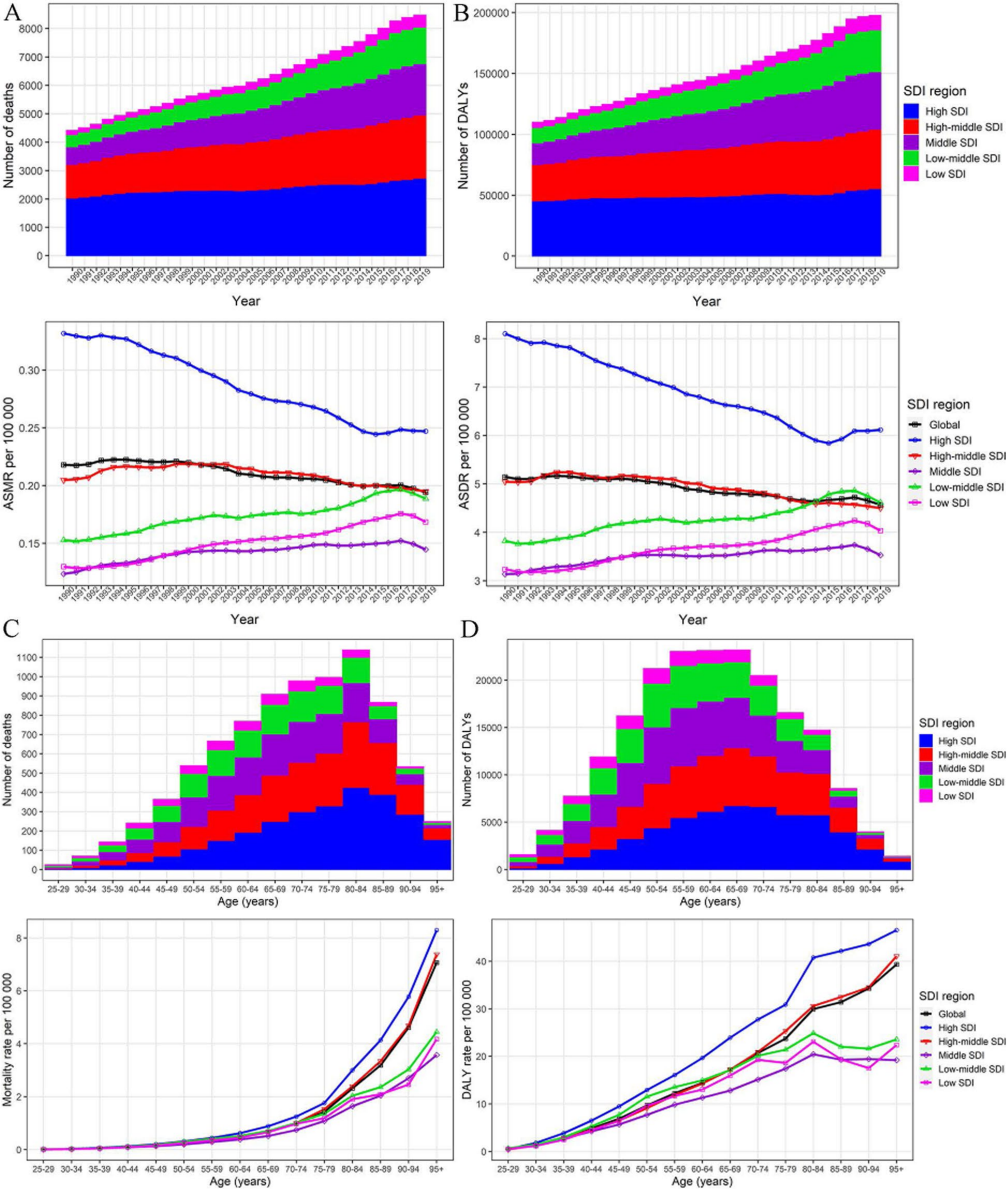
The breast cancer burden attributable to low physical activity by SDI region. The global (**A**) deaths and ASMR, and (**B**) DALYs and ASDR of breast cancer attributable to low physical activity for all ages from 1990 to 2019. The global (**C**) deaths and age-specific mortality rate, and (**D**) DALYs and age-specific DALY rate of breast cancer attributable to low physical activity by age in 2019. SDI, Socio-demographic Index; ASMR, age-standardized mortality rate; DALYs, disability-adjusted life years; ASDR, age-standardized DALY rate

The deaths of LPA-related breast cancer increased by nearly 92 percent from 4,412 (95% *UI*: 2,006, 7,693) to 8,475 (95% *UI*: 4,078, 14,305), while the ASMR decreased from 0.22 (95% *UI*: 0.10, 0.38) to 0.19 (95% *UI*: 0.09, 0.33) per 100,000, with EAPC of -0.46 (95% *CI*: -0.52, -0.40) worldwide from 1990 to 2019 (Table 1, Fig. 1A).

**Table 1:** The global breast cancer burden attributable to low physical activity in 1990 and 2019 and the temporal trends from 1990 to 2019

Characteristics	1990				2019				EAPC (1990–2019)	
	**Deaths cases**	**ASMR per 10**^**5**^	**DALYs**	**ASDR per 10**^**5**^	**Deaths cases**	**ASMR per 10**^**5**^	**DALYs**	**ASDR per 10**^**5**^	**ASMR**	**ASDR**
	**No. (95% *UI*)**	**No. (95% *UI*)**	**No. × 10**^**3**^** (95% *UI*)**	**No. (95% *UI*)**	**No. (95% *UI*)**	**No. (95% *UI*)**	**No. × 10**^**3**^** (95% *UI*)**	**No. (95% *UI*)**	**No. (95% *CI*)**	**No. (95% *CI*)**
**Overall**	4412 (2006, 7693)	0.22 (0.10, 0.38)	109.95 (51.95, 202.20)	5.14 (2.43, 9.38)	8475 (4078, 14,305)	0.19 (0.09, 0.33)	197.80 (97.52, 345.14)	4.57 (2.26, 7.98)	-0.46 (-0.52, -0.40)	-0.44 (-0.49, -0.39)
**SDI region**										
High SDI	2036 (755, 3768)	0.33 (0.13, 0.63)	45.40 (17.20, 88.69)	8.10 (3.14, 16.08)	2733 (994, 4932)	0.25 (0.09, 0.45)	55.55 (21.13, 103.29)	6.12 (2.3, 11.75)	-1.21 (-1.28, -1.14)	-1.17 (-1.25, -1.08)
High-middle SDI	1184 (571, 1995)	0.20 (0.10, 0.34)	29.84 (14.72, 51.22)	5.05 (2.50, 8.74)	2227 (1038, 3714)	0.19 (0.09, 0.32)	48.78 (23.48, 82.28)	4.50 (2.21, 7.69)	-0.28 (-0.40, -0.16)	-0.51 (-0.60, -0.42)
Middle SDI	621 (331, 1075)	0.12 (0.06, 0.21)	17.93 (9.95, 32.03)	3.14 (1.71, 5.56)	1806 (932, 3039)	0.14 (0.07, 0.24)	47.17 (24.94, 83.77)	3.53 (1.87, 6.27)	0.59 (0.48, 0.69)	0.49 (0.40, 0.58)
Low-middle SDI	426 (217, 729)	0.15 (0.08, 0.25)	12.47 (6.74, 22.02)	3.82 (2.01, 6.59)	1283 (677, 2098)	0.19 (0.10, 0.31)	34.34 (18.98, 58.8)	4.61 (2.51, 7.83)	0.84 (0.76, 0.92)	0.81 (0.72, 0.91)
Low SDI	142 (76, 246)	0.13 (0.07, 0.22)	4.21 (2.35, 7.60)	3.24 (1.76, 5.70)	41 (232, 711)	0.17 (0.09, 0.28)	11.78 (6.74, 20.81)	4.03 (2.25, 6.91)	1.14 (1.07, 1.22)	1.02 (0.94, 1.10)
**GBD region**										
High-income Asia Pacific	104 (40, 214)	0.09 (0.04, 0.19)	2.95 (1.20, 6.43)	2.68 (1.10, 5.86)	298 (107, 567)	0.12 (0.04, 0.24)	6.28 (2.35, 12.85)	3.21 (1.26, 6.99)	1.00 (0.87, 1.14)	0.83 (0.69, 0.98)
High-income North America	733 (259, 1426)	0.36 (0.13, 0.70)	17.22 (6.32, 35.14)	9.30 (3.41, 19.47)	778 (285, 1462)	0.22 (0.08, 0.42)	17.44 (6.34, 34.73)	5.69 (2.08, 11.64)	-1.84 (-1.99, -1.68)	-1.85 (-1.93, -1.76)
Western Europe	1495 (527, 2701)	0.44 (0.16, 0.81)	32.02 (11.75, 60.80)	10.67 (3.89, 20.58)	1992 (667, 3562)	0.35 (0.12, 0.64)	36.15 (12.79, 66.22)	8.10 (2.8, 15.39)	-1.05 (-1.18, -0.92)	-1.31 (-1.48, -1.14)
Australasia	47 (17, 90)	0.37 (0.13, 0.70)	1.09 (0.40, 2.16)	8.88 (3.28, 17.87)	91 (29, 165)	0.33 (0.11, 0.60)	1.93 (0.65, 3.58)	8.03 (2.63, 15.39)	-0.45 (-0.51, -0.39)	-0.37 (-0.44, -0.31)
Southern Latin America	36 (22, 73)	0.14 (0.09, 0.29)	0.92 (0.57, 1.96)	3.68 (2.28, 7.89)	72 (40, 135)	0.15 (0.08, 0.28)	1.58 (0.90, 3.04)	3.58 (2.07, 7.03)	0.20 (0.05, 0.36)	-0.03 (-0.14, 0.08)
Andean Latin America	6 (4, 14)	0.06 (0.04, 0.13)	0.18 (0.12, 0.49)	1.48 (0.99, 3.96)	19 (11, 41)	0.07 (0.04, 0.14)	0.48 (0.29, 1.15)	1.61 (0.96, 3.76)	0.39 (0.27, 0.51)	0.08 (-0.04, 0.19)
Tropical Latin America	226 (78, 394)	0.47 (0.17, 0.81)	6.59 (2.21, 11.75)	12.33 (4.14, 21.91)	594 (220, 1004)	0.45 (0.16, 0.75)	15.35 (5.42, 26.24)	11.48 (4.06, 19.58)	-0.21 (-0.39, -0.03)	-0.24 (-0.43, -0.05)
Central Latin America	44 (21, 86)	0.10 (0.05, 0.20)	1.30 (0.63, 2.67)	2.66 (1.29, 5.44)	138 (63, 272)	0.11 (0.05, 0.22)	3.65 (1.74, 7.44)	2.79 (1.33, 5.64)	0.31 (0.20, 0.42)	0.42 (0.30, 0.54)
Caribbean	45 (16, 82)	0.34 (0.12, 0.62)	1.12 (0.43, 2.14)	8.19 (3.08, 15.60)	109 (38, 199)	0.39 (0.14, 0.71)	2.54 (0.92, 4.82)	9.33 (3.38, 17.77)	0.63 (0.55, 0.72)	0.66 (0.58, 0.74)
Eastern Europe	215 (132, 364)	0.12 (0.08, 0.21)	5.44 (3.43, 9.89)	3.28 (2.09, 6.13)	325 (184, 543)	0.15 (0.08, 0.25)	6.91 (4.06, 12.21)	3.43 (2.07, 6.21)	0.42 (0.16, 0.68)	-0.09 (-0.33, 0.16)
Central Europe	187 (101, 303)	0.23 (0.13, 0.38)	4.45 (2.48, 7.61)	5.55 (3.12, 9.57)	320 (161, 531)	0.25 (0.13, 0.41)	6.24 (3.27, 10.47)	5.52 (2.95, 9.41)	0.22 (0.12, 0.32)	-0.03 (-0.12, 0.06)
Central Asia	36 (22, 61)	0.13 (0.08, 0.22)	0.96 (0.61, 1.80)	3.47 (2.22, 6.49)	55 (33, 97)	0.14 (0.09, 0.25)	1.48 (0.91, 2.78)	3.40 (2.07, 6.19)	0.55 (0.42, 0.68)	0.01 (-0.10, 0.11)
North Africa and Middle East	183 (75, 326)	0.22 (0.09, 0.38)	5.53 (2.35, 10.19)	5.80 (2.44, 10.53)	680 (272, 1201)	0.32 (0.13, 0.55)	20.32 (7.94, 36.49)	8.45 (3.32, 15.14)	1.44 (1.34, 1.53)	1.34 (1.27, 1.41)
South Asia	400 (201, 685)	0.17 (0.08, 0.28)	11.49 (5.94, 20.56)	3.92 (2.01, 6.82)	1246 (667, 2051)	0.19 (0.10, 0.31)	31.60 (17.66, 54.57)	4.30 (2.37, 7.3)	0.79 (0.60, 0.99)	0.89 (0.69, 1.10)
Southeast Asia	153 (92, 304)	0.11 (0.07, 0.21)	4.79 (3.00, 10.11)	3.06 (1.88, 6.25)	408 (233, 754)	0.13 (0.07, 0.23)	11.55 (6.79, 22.93)	3.31 (1.93, 6.48)	0.31 (0.24, 0.39)	0.15 (0.09, 0.21)
East Asia	347 (175, 621)	0.08 (0.04, 0.15)	9.55 (5.17, 17.92)	2.03 (1.08, 3.71)	889 (448, 1590)	0.08 (0.04, 0.15)	21.83 (11.52, 40.93)	2.03 (1.07, 3.8)	-0.32 (-0.46, -0.19)	-0.43 (-0.58, -0.27)
Oceania	6 (2, 12)	0.42 (0.17, 0.81)	0.20 (0.08, 0.42)	11.67 (4.67, 23.26)	21 (8, 41)	0.58 (0.22, 1.10)	0.68 (0.27, 1.41)	15.93 (6.08, 32.04)	1.03 (0.99, 1.06)	1.03 (0.98, 1.08)
Western Sub-Saharan Africa	62 (33, 111)	0.15 (0.08, 0.27)	1.68 (0.91, 3.04)	3.67 (1.98, 6.64)	201 (102, 360)	0.22 (0.11, 0.40)	5.58 (2.88, 10.14)	5.13 (2.58, 9.12)	1.33 (1.29, 1.38)	1.19 (1.12, 1.26)
Eastern Sub-Saharan Africa	26 (17, 58)	0.07 (0.05, 0.15)	0.81 (0.52, 1.95)	1.84 (1.19, 4.19)	73 (47, 154)	0.09 (0.06, 0.18)	2.09 (1.37, 4.92)	2.14 (1.39, 4.67)	0.82 (0.72, 0.92)	0.50 (0.37, 0.62)
Central Sub-Saharan Africa	22 (10, 41)	0.21 (0.10, 0.39)	0.65 (0.31, 1.26)	4.95 (2.33, 9.27)	73 (34, 141)	0.29 (0.13, 0.53)	2.01 (0.93, 3.89)	6.39 (2.98, 12.23)	0.91 (0.71, 1.12)	0.78 (0.58, 0.98)
Southern Sub-Saharan Africa	38 (16, 69)	0.25 (0.11, 0.46)	1.01 (0.45, 1.92)	6.06 (2.69, 11.29)	91 (42, 160)	0.30 (0.14, 0.53)	2.12 (0.99, 3.82)	6.39 (3.01, 11.37)	1.27 (0.82, 1.73)	0.86 (0.43, 1.29)

*No.* number, *ASMR* age-standardized mortality rate, *UI* uncertainty interval, *DALYs* disability-adjusted life years, *ASDR* age-standardized DALY rate, *EAPC* estimated annual percentage change, *CI* confidence interval, *SDI* Socio-demographic Index

At the SDI-regional level, the LPA-related breast cancer deaths mainly occurred in high and high-middle SDI regions, where the ASMR decreased during this period, with EAPC of -1.21 (95% *CI*: -1.28, -1.14) and -0.28 (95% *CI*: -0.40, -0.16), respectively. The middle, low-middle and low SDI regions had significant increases in ASMR, with EAPC ranging from 0.59 to 1.14 (Table 1, Fig. 1A).

At the GBD-regional level, the LPA-related breast cancer deaths mainly occurred in Western Europe and High-income North America, which accounted for 50.51% of the global deaths in 1990, and Western Europe, South Asia and East Asia became the top three countries in 2019. From 1990 to 2019, there were downward trends in ASMR in High-income North America, Western Europe, Australasia, Tropical Latin America and East Asia, with the EAPC ranging from -1.84 to -0.21, whereas ASMRs of the other GBD regions were upward, with the fastest growth observed in North Africa and Middle East (EAPC, 1.44; 95% *UI*: 1.34, 1.53; Table 1).

**Fig. 2:**
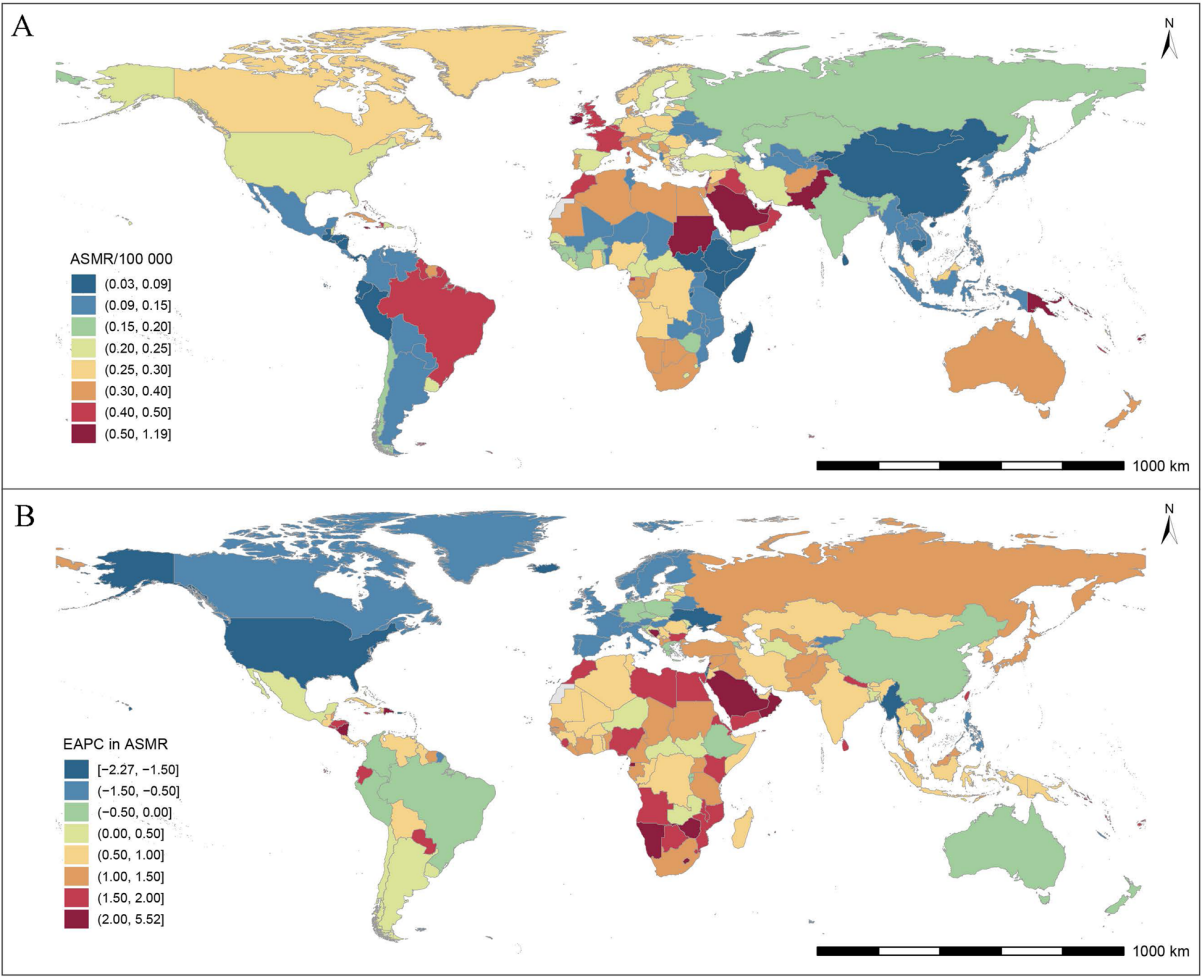
The spatial distribution of breast cancer (**A**) ASMR and (**B**) the EAPC attributable to low physical activity in 2019. ASMR, age-standardized mortality rate; EAPC, estimated annual percentage change

At the national level, the USA had the most breast cancer deaths attributable to LPA in 1990 (669; 95% *UI*: 236, 1298), while India and China became the largest contributions in 2019 (873 and 848, respectively). The highest ASMR was observed in Malta in 1990 (0.96 per 100,000; 95% *UI*: 0.33, 1.64) and Qatar in 2019 (1.19 per 100,000; 95% *UI*: 0.46, 2.06), whereas Guatemala always had the lowest ASMR during this period (0.04 in 1990, 0.03 in 2019, per 100,000). There were 143 countries and territories with upward ASMR, with the fastest increase observed in Solomon Islands (EAPC, 5.52; 95% *CI*: 4.88, 6.17; Fig. 2, Additional Table 1).

In 2019, the number of LPA-related breast cancer deaths showed an inverted V-shaped association with age, peaking at the 80–84 age group, but the age-specific mortality rate increased exponentially with age globally or in all SDI regions. The deaths mainly occurred in individuals of 65–89 years old, the majority of which were in high and high-middle SDI regions (Fig. 1C). Globally, the EAPC in mortality rates showed a W-shaped association with age, with the value below 0 in individuals of 30–89 years and above 0 in 25–29 and 95 + age groups, and the fastest decrease in mortality rates occurred in 45–49 age group, and the fastest increase occurred in 95 + age group. However, the age-specific mortality rates in low-middle and low SDI regions were in upward trends for all age groups (Additional Fig. 1).

### Breast cancer DALYs and ASDR attributable to LPA

From 1990 to 2019, globally, the number of LPA-related breast cancer DALYs increased by about 80 percent from 109.95 (95% *UI*: 51.95, 202.20) thousand to 197.80 (95% *UI*: 97.52, 345.14) thousand, but the ASDR decreased from 5.14 (95% *UI*: 2.43, 9.38) to 4.57 (95% *UI*: 2.26, 7.98) per 100,000, with EAPC of -0.44 (95% *CI*: -0.49, -0.39; Table 1, Fig. 1B).

At the SDI-regional level, high and high-middle SDI regions always had more than half of the global DALYs, although the corresponding ASDR was in a downward trend, with EAPC of -1.21 (95% *CI*: -1.28, -1.14) and -1.17 (95% *CI*: -1.25, -1.08), respectively. The DALYs and ASDR in middle, low-middle and low SDI regions were all in upward trends, with the fastest increase in ASDR occurring in low SDI regions (EAPC = 1.02; 95% *CI*: 0.94, 1.10; Table 1, Fig. 1B).

At the GBD-regional level, the regions with DALYs over 20 thousand were Western Europe, South Asia and East Asia in 2019 (36.15, 31.60 and 21.83 thousand, respectively), but only Western Europe in 1990 (32.02 thousand). However, the highest ASDR was observed in Tropical Latin America in 1990 (12.33 per 100,000; 95% *UI*: 4.14, 21.91), and in Oceania in 2019 (15.93 per 100,000; 95% *UI*: 6.08, 32.04). The ASDRs were downward in High-income North America, Western Europe, Australasia, Tropical Latin America and East Asia, with EAPC ranging from -1.85 to -0.24, pretty stable in Eastern Europe, Central Europe, Central Asia, Southern Latin America and Andean Latin America, and upward in the other GBD regions, with EAPC ranging from 0.15 to 1.34 (Table 1).

**Fig. 3:**
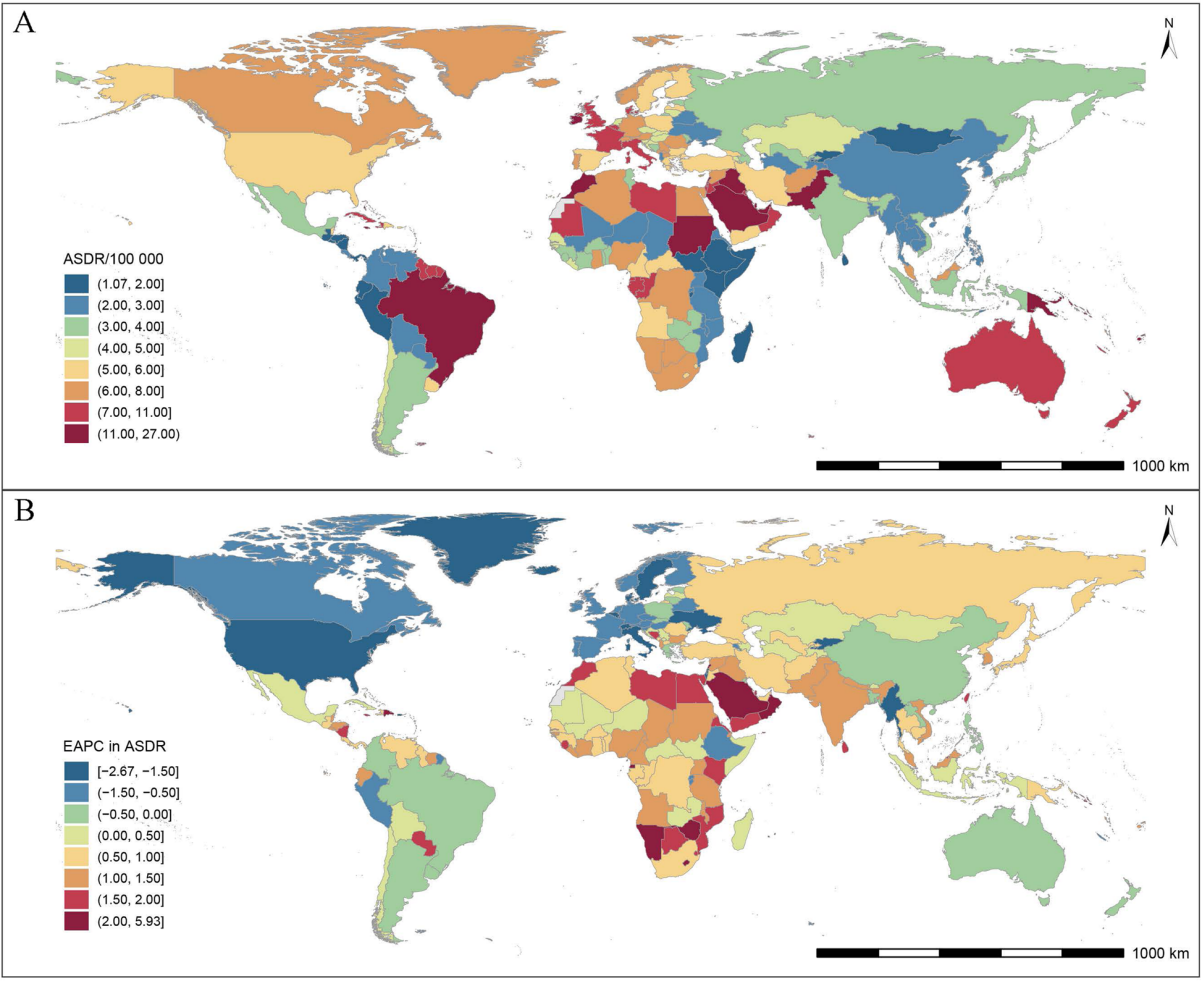
The spatial distribution of breast cancer (**A**) ASDR and (**B**) the EAPC attributable to low physical activity in 2019. ASDR, age-standardized disability-adjusted life-years rate; EAPC, estimated annual percentage change

At the national level, the USA had the most DALYs (15.76 thousand; 95% *UI*: 5.76, 32.10) in 1990, while India and China became the top 2 countries with the most DALYs (21.27 and 20.81 thousand, respectively) in 2019. The highest ASDR was observed in Malta (24.10 per 100,000; 95% *CI*: 7.51, 42.50) in 1990 and Solomon Islands (26.96 per 100,000; 95% *CI*: 11.20, 56.56) in 2019, and the lowest ASDR was always observed in Guatemala during this period (0.88 in 1990 and 1.07 in 2019 per 100,000). There were 135 countries and territories with upward ASDR from 1990 to 2019, and 46 countries and territories with downward ASDR (Additional Table 1, Fig. 3).

In 2019, the age-specific numbers of LPA-related breast cancer DALYs showed a similar pattern with that of deaths, however peaking in individuals of 55–69 years old. The age-specific DALY rates had a sustained increase with age in high and high-middle SDI regions, however, which increased first before 80–84 years old and then fluctuated slightly with age in the middle, low-middle and low SDI regions (Fig. 1D). Besides, the association between EAPC in DALY rate and age was similar to that between EAPC in mortality rate and age (Additional Fig. 2).

### The changes in physical activity levels

Meanwhile, the global proportion of inactive cases was essentially flat, and that of low-active cases slightly increased from 1990 to 2019. Oceania, High-income Asia Pacific, Australasia, Western Europe, Caribbean, Tropical Latin America, and North Africa and Middle East had a constantly higher proportion of inactive and low-active cases than the globe (Additional Fig. 3–4). However, the proportion of moderately and highly active cases had a slight decline across the world during the last 3 decades, which in Western Europe, High-income North America, Caribbean, and North Africa and Middle East were lower than the global average proportion (Additional Fig. 5).

**Fig. 4:**
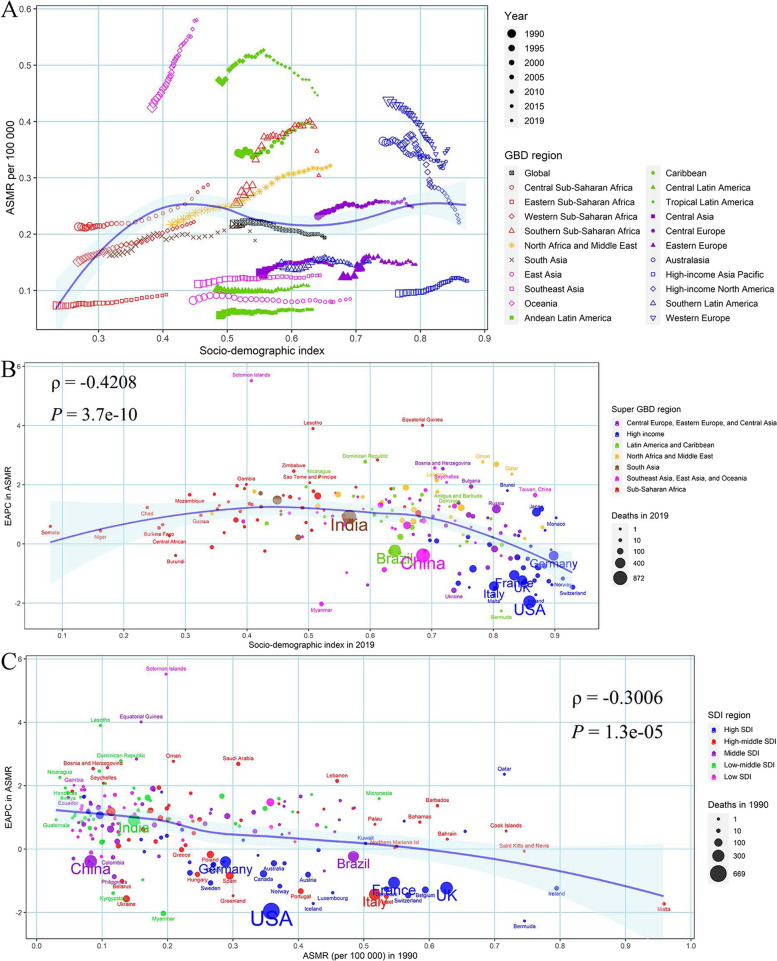
The relationship between breast cancer (**A**) ASMR and SDI in 2019 by GBD region, (**B**) EAPC in ASMR and SDI in 2019 by Super GBD region, and (**C**) EAPC in ASMR and ASMR in 1990 by SDI region. ASMR, age-standardized mortality rate; SDI, Socio-demographic Index; GBD, Global Burden of Disease Study; EAPC, estimated annual percentage change

**Fig. 5:**
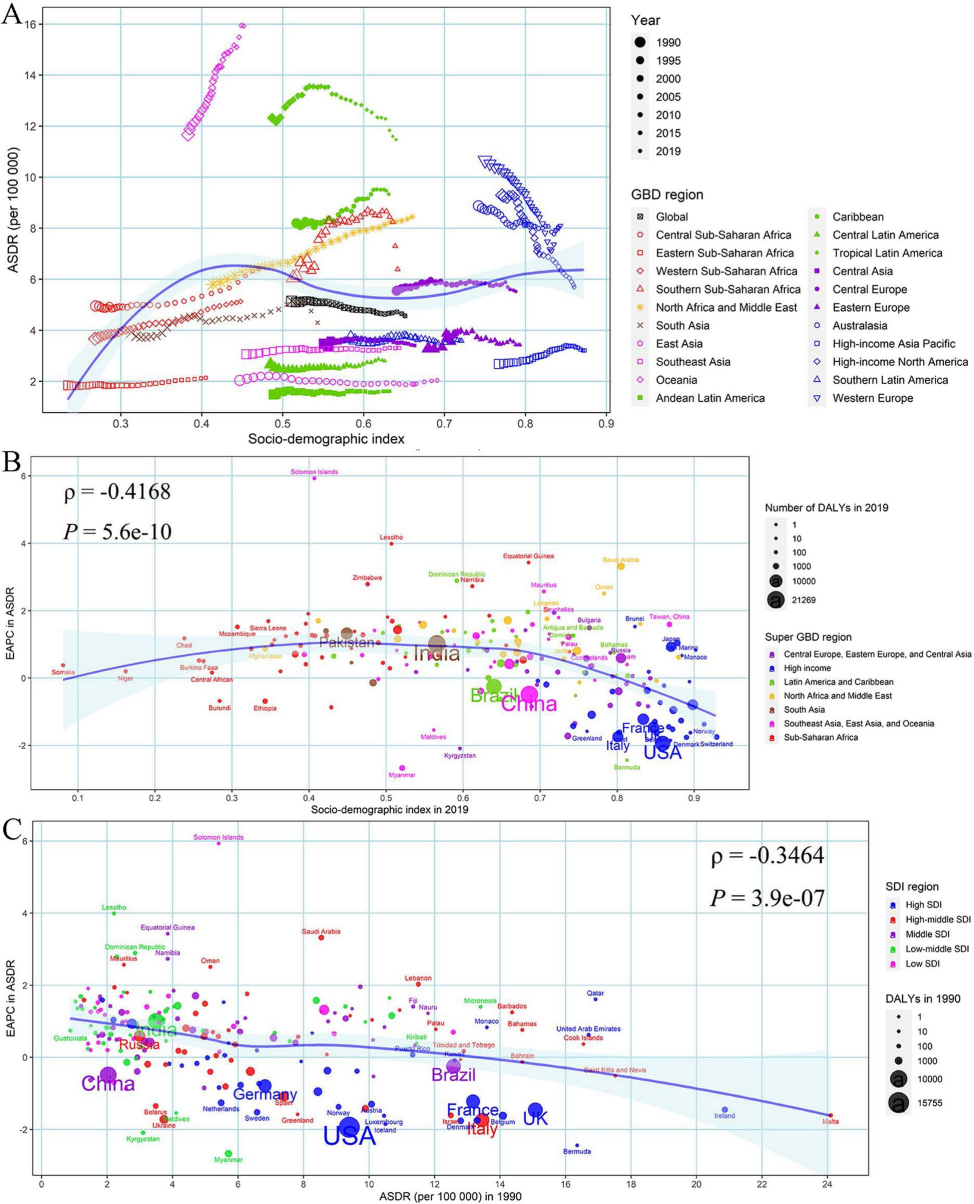
The relationship between breast cancer (**A**) ASDR and SDI in 2019, (**B**) EAPC in ASDR and SDI in 2019, and (**C**) EAPC in ASDR and ASDR in 1990. ASDR, age-standardized disability-adjusted life-years rate; SDI, Socio-demographic Index; EAPC, estimated annual percentage change

### Association between ASMR, ASDR of breast cancer and SDI values

There was a logarithmic association between ASMR of LPA-related breast cancer and SDI values by GBD region, that the ASMR increased first until SDI value was about 0.4, and kept relatively stable for higher SDI values (Fig. 4A). The EAPC in ASMR of LPA-related breast cancer showed a slightly negative association with SDI values in 2019 (*ρ* = -0.42, *P* < 0.001) especially within SDI values greater than 0.5, and with ASMR in 1990 (*ρ* = -0.30, *P* < 0.001), although the ASMR in Solomon Islands, Equatorial Guinea and Lesotho was much higher than expected (Fig. 4B-C). The ASMR presented an upward trend from 1990 to 2019 mainly in most of 204 countries (the EAPC with its 95% *CI* higher than 0), such as Solomon Islands, Equatorial Guinea, Japan and India, but in downward trends in some countries like Bermuda, Myanmar, USA and China, regardless of their SDI status or ASMR in 1990 (Additional Table 1, Fig. 4B-C). There were similar patterns between ASDR, SDI and EAPC in ASDR (Fig. 5).

## Discussion

In this study, spatiotemporal trends in deaths and DALYs of LPA-related breast cancer were estimated at global, regional and national levels. Globally, from 1990 to 2019, both LPA-related breast cancer deaths and DALYs nearly doubled, but the corresponding ASMR and ASDR decreased slightly. The LPA-related breast cancer burden varied considerably across the world, with the high-burden rates in Oceania, Tropical Latin America and Caribbean, and the fastest growth in North Africa and Middle East. There was a logarithmic association between LPA-related breast cancer burden and SDI values, with the severest burden observed in high and high-middle SDI regions. The ASMR and ASDR increased in most of the 204 countries and territories from 1990 to 2019, such as Solomon Islands, Equatorial Guinea, Japan and India, but the declines were achieved in some countries like Bermuda, Myanmar, USA and China.

Since the 1980s, it has been generally assumed that physical activity is associated with decreased risk of breast cancer, with many studies conducted to demonstrate this assumption during the past 4 decades [15, 16]. The 2018 US Physical Activity Guidelines concluded strong evidence that more physical activity reduced not only the risk of breast cancer but also the risk of mortality from breast cancer [17]. Wu et al*.* reported a dose–response association that the risk of breast cancer decreased by 2% with the increase in non-occupational physical activity of every 25 MET-hours per week [16]. Nevertheless, the exact pathophysiological mechanisms for physical activity against the breast cancer risk have not been known clearly. Several reviews, on one hand, indicated that obesity was an identified risk factor of breast cancer, and physical inactivity usually contributed to obesity [18, 19], but Moore et al*.* conducted a meta-analysis and found that the hazard ratio for breast cancer risk in individuals with high *vs* low levels of physical activity was 0.90 (95% *CI*, 0.80–0.95), and remained the same trend after body mass index adjustment [20]. On the other hand, the efficacy of physical activity to decrease sex hormone levels [21], lower insulin levels and insulin resistance [22], and reduce systemic inflammation [23], has been demonstrated to prevent the development of breast cancer [24], which may help to explain why physical activity reduces the risk of breast cancer, regardless of the body weight.

In our study, the age-specific rates of mortality and DALYs increased with age, and the LPA-related breast cancer burden was heavier in the elderly than the young, which could mostly represent a high level of LPA in the elderly. In General, keeping physically active is beneficial to physical capability and counteracting the effects of ageing for the elderly [25]. A prospective cohort study conducted in individuals from midlife to old age indicated that the moderate-increasing pattern of physical activity during this period could provide significant protection against cardiovascular diseases, as well as the all-cause mortality [26], which might be due to the direct amelioration of immune aging [27]. However, there was a dramatic fall of physical activity with age [27], and the hormone replacement therapy among postmenopausal women could nullify the protection of physical activity [15]. In recent years, the survival rates and life expectancy in the elderly continue to increase, furthering the global ageing trends [28], and the LPA-related breast cancer burden in the elderly. Therefore, facilitating physical activity is particularly essential for the elderly, which needs strengthened efforts and policy support.

LPA-related breast cancer burden varied across the world, which indicated the global changes in physical activity levels. From 1990 to 2019, the global levels of physical activity, especially the level of moderate and high physical activity, were in slightly downward trends, and it was the same when analyzed by GBD regions. And this was consistent with our results of the temporal increase of LPA-related breast cancer burden from 1990 to 2019 observed in the lower SDI regions. With the industrialization and development of the economy, individuals in lower SDI regions would take up jobs with fewer demands on physical activity, and engage in less leisure-time physical activity, due to changes in transport patterns, entertainment and cultural values [29, 30], which meant that the total physical activity in lower SDI regions shifted towards a low level. On the other hand, it was noteworthy that the current values in some lower- and middle-income countries were possibly underestimated, where the cancer surveillance systems started relatively late. The National Central Cancer Registry of China, for instance, was established in 2002, and the number of local registries increased to 501 by August 2018, which covered 387 million population [31]. Published studies estimated the breast cancer burden in China based on the high-quality parts of total registries, which could only involve about 10% of the total population [31, 32]. Besides, it was worth noting that the individuals in high socioeconomic regions, such as Western Europe and High-income North America, were significantly less physically active than the global average levels, which might be the reason for the heaviest burden occurring in these higher SDI regions. Similarly, a previous systematic review also reported the association between high socioeconomic status and low physical activity levels [33]. Nevertheless, the LPA-related breast cancer burden declined in high and high-middle SDI regions from 1990 to 2019, probably owing to the earlier focus on physical activity surveillance and intervention to promote physical activity in high-income countries [34]. It illustrated that local government especially in the lower-SDI countries, should take examples from the higher-SDI countries and put more efforts into promoting physical activity, and breast cancer surveillance and control.

Despite the significant epidemiological and clinical research progress achieved which promotes a better understanding of the protective effects of physical activity, the LPA-related breast cancer burden, far from showing a downward trend, has been increasing during the last 3 decades. This analysis measures the effectiveness of previous interventions in such a way and indicates that there is an urgent need for strengthening the surveillance of physical activity and implementing the appropriate public health strategies, to mobilize populations for keeping physically active, especially for the countries with increasing LPA-related breast cancer burden. For example, China is one of the countries with the most LPA-related breast cancer burden, and the Chinese government recently starts to invest heavily in the supply of community fitness facilities, and motivate their people to take part in physical exercises or competitions, which could truly promote the national level of physical activity, to control the breast cancer burden. Currently, WHO guidelines suggest at least 150–300 min per week of moderate-intensity aerobic physical activity for adults and an average of 60 min per day of moderate- to vigorous-intensity physical activity for children and adolescents [35], which seems to be generally realizable at the populational level. However, there is still a long way to go to achieve this goal, which means the stakeholders should develop suitable strategies to promote physical activity levels for their countries.

However, this analysis does have some limitations. First, the GBD study measured physical activity based on the previous epidemiological studies and national health-survey databases [11], which might lead to insufficient available data in low- and middle-income countries. The whole data were ecological and cross-sectional, and could not provide the exposure levels of physical activity and our study outcomes on the same people, which might have little impact on the reliability of our results, for the comparative risk assessment framework included the causal attribution steps based on the previous primary studies or secondary meta-analysis. Second, the GBD study identified a total of six risk factors for breast cancer, including high fasting plasma glucose, alcohol use, high BMI, diet high in red meat, low physical activity, and tobacco (smoking, chewing tobacco and secondhand smoke), but didn’t include the pathological data of breast cancer, and only provided data on LPA-related breast cancer burden among females, and thus we didn’t include subtypes of breast cancer and sex into the analysis. Finally, there might be some uncertainty about the estimates of physical activity and changes in MET-minutes per week over time, because the data were derived from self-reported questionnaires and there was no supplementary data to verify the truthful levels.

## Conclusions

In summary, although the LPA-related breast cancer burden rates decreased globally from 1990 to 2019, there was still an increase occurring in most countries or territories, such as Solomon Islands, Equatorial Guinea, Japan and India, which, to a certain extent, was owing to the increasing levels of physical inactivity. Our findings can bring greater awareness to the importance of promoting physical activity in the public for breast cancer burden control, and urge the policymakers to develop strategies that are easy to implement and allocate sports resources equitably.

## Supplementary Information

**Additional file 1.**

**Additional file 2.**

**Additional file 3.**

## Data Availability

All data could be extracted from the Global Health Data Exchange (GHDx) website (http://ghdx.healthdata.org/gbd-results-tool).

## Abbreviations

ASDR: Age-standardized disability-adjusted life-year rate
ASMR: Age-standardized mortality rate
ASR: Age-standardized rate
CI: Confidence interval
DALY: Disability-adjusted life year
EAPC: Estimated annual percentage change
GBD: Global Burden of Disease Study
LPA: Low physical activity
MET: Metabolic Equivalent
SDI: Socio-demographic Index
UI: Uncertainty interval
