# Supplementary material for: The global, regional, and national disease burden of breast cancer attributable to low physical activity from 1990 to 2019: an analysis of the Global Burden of Disease Study 2019
Source: Int J Behav Nutr Phys Act. 2022 Apr 2;19:42. doi: 10.1186/s12966-022-01283-3 (PMC8977046; doi:10.1186/s12966-022-01283-3)
Supplement: Supplementary file 3 — Additional file 3. [file 12966_2022_1283_MOESM3_ESM.docx]

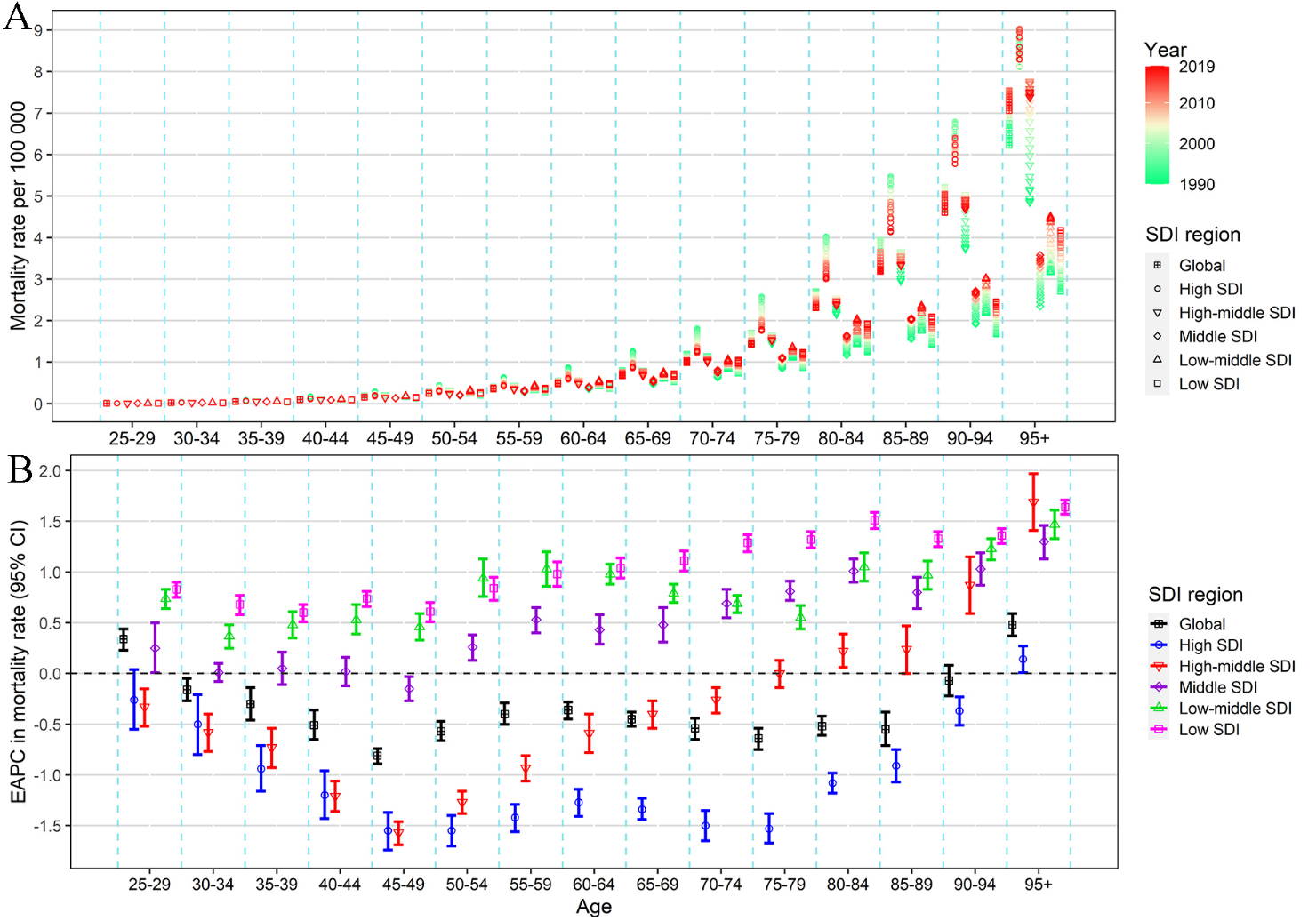
Additional Fig. 1 The age distribution of (A) age-specific mortality rate and (B) EAPC in age-specific mortality rate attributable to low physical activity by SDI region from 1990 to 2019. EAPC, estimated annual percentage change; SDI, Socio-demographic Index.


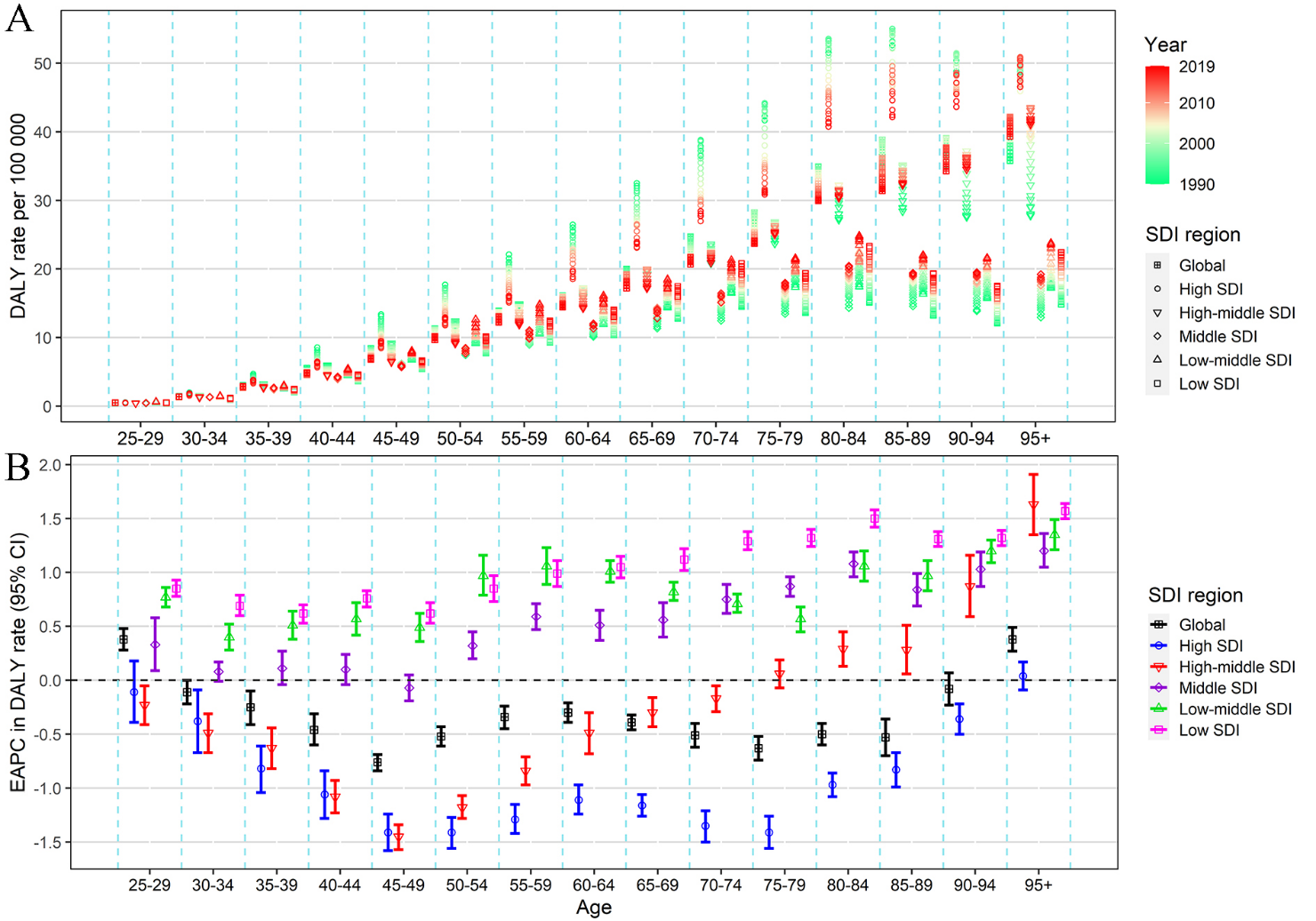
Additional Fig. 2 The age distribution of (A) age-specific DALY rate and (B) EAPC in age-specific DALY rate attributable to low physical activity by SDI region from 1990 to 2019. DALYs, disability-adjusted life year; EAPC, estimated annual percentage change; SDI, Socio-demographic Index.


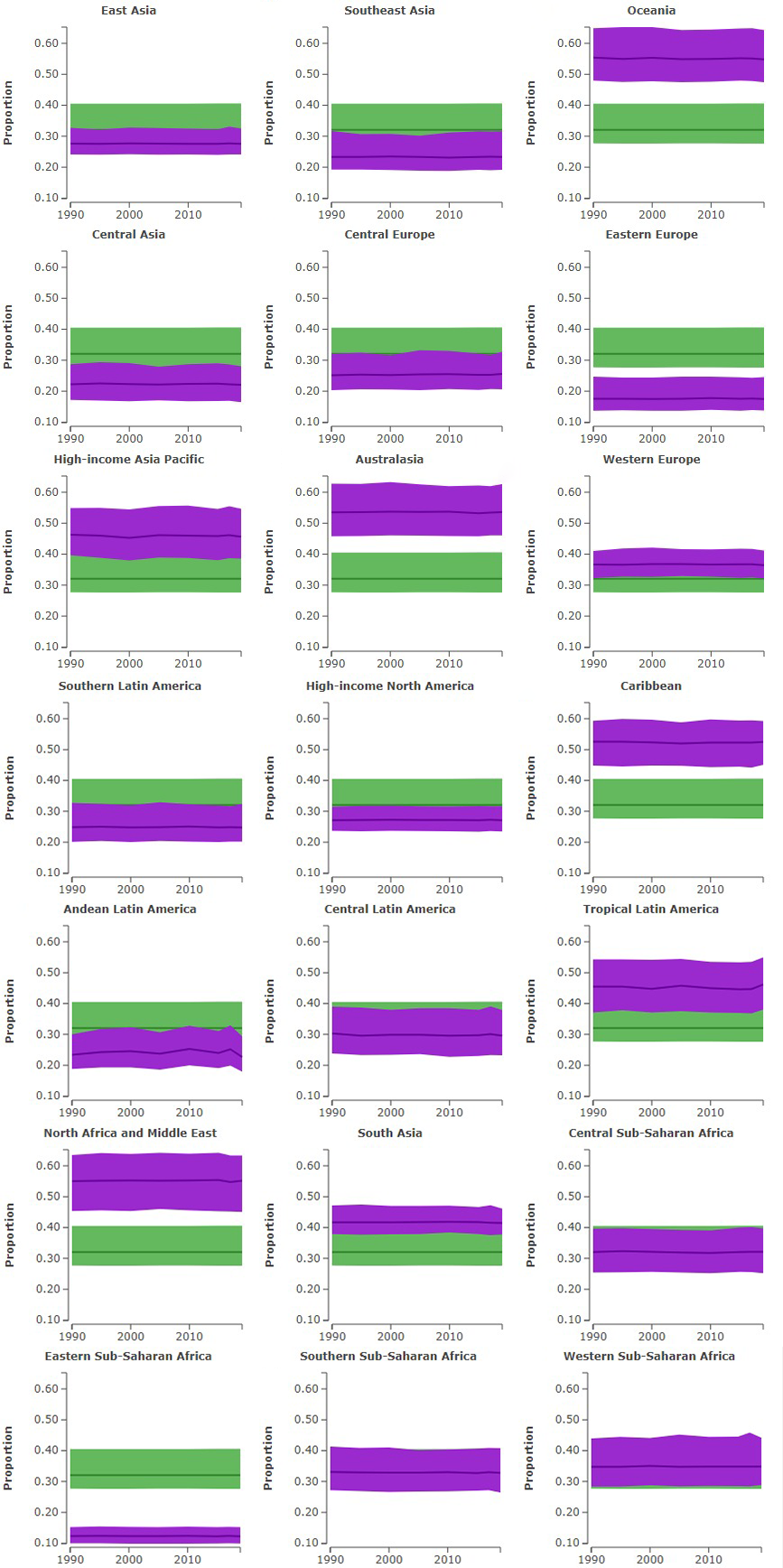


Additional Fig. 3 The proportion of inactive cases of 21 GBD regions from 1990 to 2019. Inactive, <600 MET-minutes per week. Proportion, the number of inactive cases in a specific population. The green indicates the global fits, i.e. the initial estimates fit to all the world’s data with estimation of the coefficients to crosswalk data with non-reference study characteristics. The purple indicates the GBD-regional fits. GBD, global burden of disease; MET, Metabolic Equivalent.


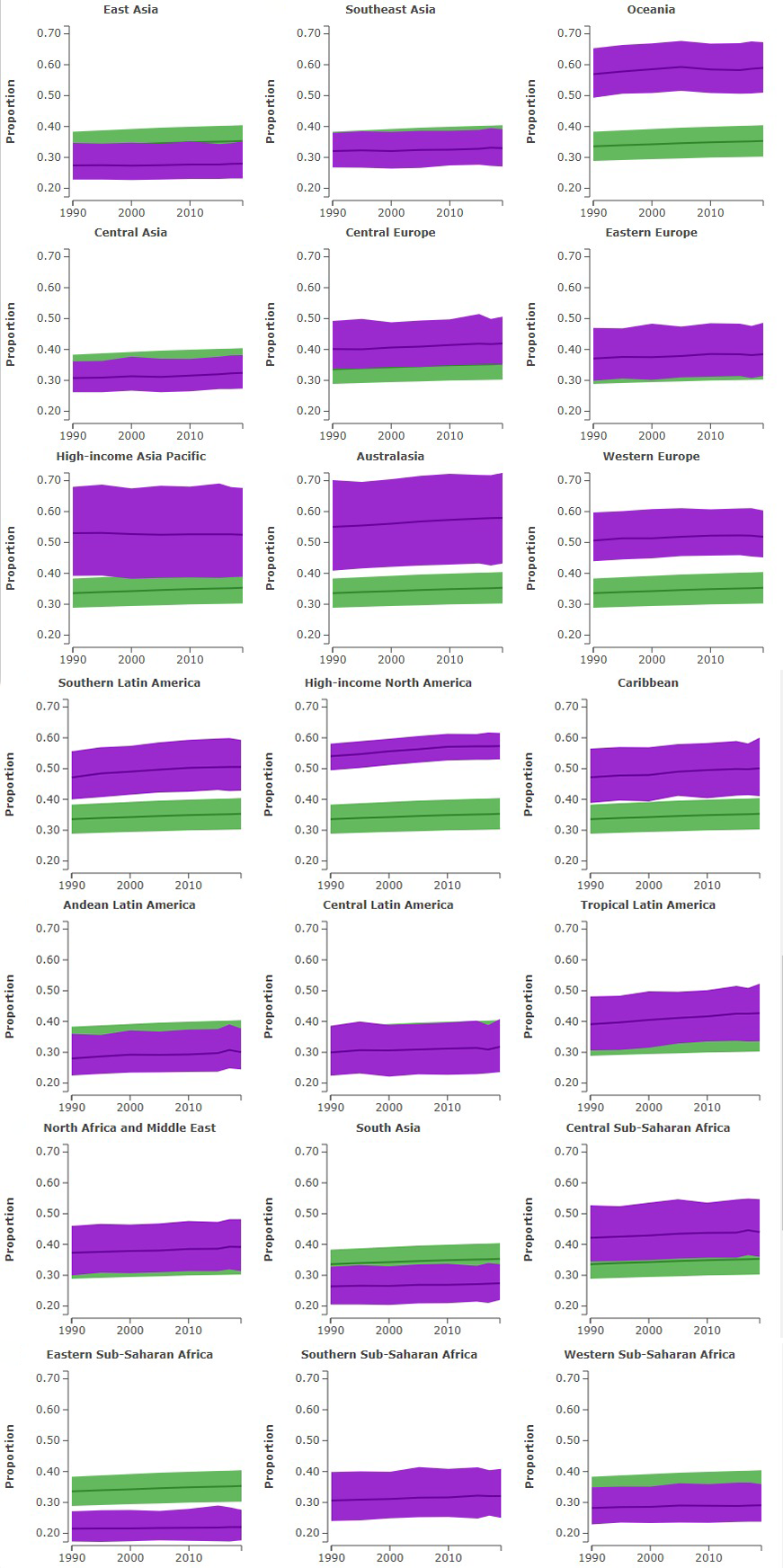


Additional Fig. 4 The proportion of low-active cases of 21 GBD regions from 1990 to 2019. Low active, 600-3999 MET-minutes per week. Proportion, the number of low active cases in a specific population. The green indicates the global fits, i.e. the initial estimates fit to all the world’s data with estimation of the coefficients to crosswalk data with non-reference study characteristics. The purple indicates the GBD-regional fits. GBD, global burden of disease; MET, Metabolic Equivalent.


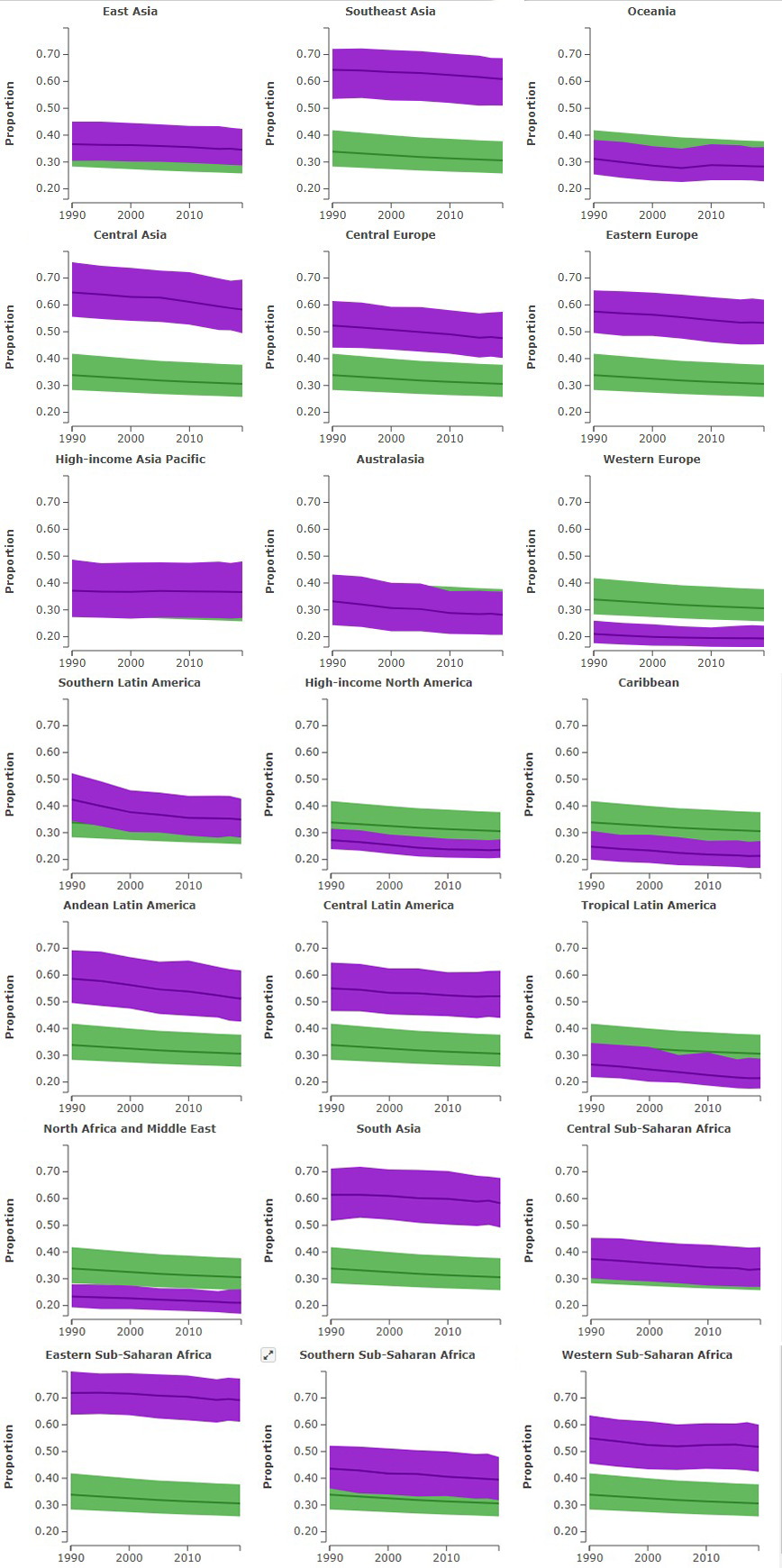


Additional Fig. 5 The proportion of moderately/highly active cases of 21 GBD regions from 1990 to 2019. Low active, >4000 MET-minutes per week. Proportion, the number of moderately/highly active cases in a specific population. The green indicates the global fits, i.e. the initial estimates fit to all the world’s data with estimation of the coefficients to crosswalk data with non-reference study characteristics. The purple indicates the GBD-regional fits. GBD, global burden of disease; MET, Metabolic Equivalent.
